# Impacts of Root Metabolites on Soil Nematodes

**DOI:** 10.3389/fpls.2019.01792

**Published:** 2020-01-31

**Authors:** Md Maniruzzaman Sikder, Mette Vestergård

**Affiliations:** ^1^ Department of Agroecology, AU-Flakkebjerg, Aarhus University, Slagelse, Denmark; ^2^ Mycology and Plant Pathology, Department of Botany, Jahangirnagar University, Dhaka, Bangladesh

**Keywords:** plant parasitic nematode, attractant, repellent, hatching stimulants, non-target organisms, signaling, nematicide, gene expression

## Abstract

Plant parasitic nematodes cause significant crop damage globally. Currently, many nematicides have been banned or are being phased out in Europe and other parts of the world because of environmental and human health concerns. Therefore, we need to focus on sustainable and alternative methods of nematode control to protect crops. Plant roots contain and release a wide range of bioactive secondary metabolites, many of which are known defense compounds. Hence, profound understanding of the root mediated interactions between plants and plant parasitic nematodes may contribute to efficient control and management of pest nematodes. In this review, we have compiled literature that documents effects of root metabolites on plant parasitic nematodes. These chemical compounds act as either nematode attractants, repellents, hatching stimulants or inhibitors. We have summarized the few studies that describe how root metabolites regulate the expression of nematode genes. As non-herbivorous nematodes contribute to decomposition, nutrient mineralization, microbial community structuring and control of herbivorous insect larvae, we also review the impact of plant metabolites on these non-target organisms.

## Introduction

Plant parasitic nematodes cause serious damage and yield losses in a wide range of crops throughout the world estimated to cause >$80 billon losses annually (Nicol et al., 2011). Due to their adverse effects on human health and the environment, chemical nematicides are being banned worldwide, and there is therefore an urgent need for alternative and efficient control measures as well as improved agricultural practices to minimize crop losses.

Functionally, root feeding nematodes are categorized according to their feeding mode. All plant feeding nematodes feed on cell solubles drawn through a stylet (in Tylenchida) or odontostyle (in Dorylaimida) pierced through the cell wall. Epidermal cell and roothair feeders are probably relatively harmless, whereas ectoparasites and endoparasites are considered harmful. Endoparasites penetrate into and feed within the root tissue, whereas ectoparasites feed exclusively from the root surface. After root penetration, female sedentary endoparasites remain at a permanent feeding site within the root for their remaining life, whereas migratory endoparasites maintain mobility and move within and between roots (Yeates et al., 1993).

A variety of plant metabolites in roots and exuded from roots to the rhizosphere influence nematode behaviour, development, reproduction and even survival (Timper et al., 2006; Dandurand and Knudsen, 2016; Wang et al., 2018). Some metabolites thus facilitate plant parasitic nematode infection and damage, whereas others directly or indirectly reduce damage. There are still a lot of mechanisms to uncover, but for some plant-nematode interactions, the molecular mechanisms are well elucidated. Profound understanding of the chemical interactions between plant roots and plant parasitic nematodes can form the basis for novel pesticide-free strategies for reduced crop damage and losses to plant parasitic nematodes.

For instance, with the identification of nematicidal root metabolites plant-breeding programs can target the development of cultivars that produce high quantities of these specific metabolites. Agricultural practices can also be adjusted to optimize plant parasitic nematode management, e.g. through the choice of crop cultivars with nematode suppressive or repellent metabolic profiles, through intelligent crop rotations that include nematode suppressive cover crops or non-susceptible crops that produce sanitizing metabolites. Detailed knowledge on chemically induced nematode egg hatching or behavior may be the basis for targeted interference with nematode host identification and infection ability. Plant metabolites may even facilitate efficient rhizosphere colonization of nematode suppressive microorganisms (Elhady et al., 2018; Topalovic et al., 2019; Topalovic and Heuer, 2019). Hence, harnessing the full potential of microbial-assisted control of plant parasitic nematodes may depend on in-depth understanding of plant chemical influence on the tripartite interactions between plants, rhizosphere microbiomes, and nematodes.

Research on plant root metabolic impacts on plant parasitic nematodes is progressing, but the research is to a large extent still scattered and some results conflicting. In order to shed clarity on the field, we compiled current knowledge on aspects of the chemical interactions between live plants and plant parasitic nematodes in agroecosystems. We aim to review thoroughly the available literature to evaluate the evidence for specific root metabolites’ impact on plant parasitic nematodes and to give an account of the different modes of action exerted by different root metabolites on plant parasitic nematodes. Further, we wish to identify gaps in current understanding and interpretation of plant chemical influence on plant parasitic nematodes. We focus on interactions between live plant roots and nematodes and refer to several excellent reviews that deal with biofumigation strategies based on cover crop soil incorporation (Fourie et al., 2016; Dutta et al., 2019). Further, we will evaluate to which extent metabolites that affect plant parasitic nematodes have unwanted effects on non-parasitic nematodes.

## Root Metabolites

About 5 to 20% of photosynthesis products are released to the rhizosphere through root exudates (Hütsch et al., 2002; Marschner, 2012). Roots deposit diverse metabolites into the rhizosphere, many of which are products of general metabolic plant processes. Deposition to the rhizosphere involves both active secretion and passive deposition of metabolites due to osmotic and concentration differences between cell and soil solutions. Several studies on model plants have identified metabolites released into the rhizosphere. For instance, 103 compounds were identified in root exudates collected from hydroponically cultivated *Arabidopsis thaliana* (Strehmel et al., 2014). In addition, root exudates of *A. thaliana* grown on MS media, were analyzed targeting primary metabolites, and 130 compounds identified (Chaparro et al., 2013). Furthermore, 289 putative secondary metabolites were quantified in *Arabidopsis* root exudates after elicitation with salicylic acid, jasmonic acid, chitosan, and two fungal cell wall elicitors (Walker et al., 2003). Chemical profiles of *Arabidopsis* thus show that a vast number of metabolites is released into the rhizosphere depending on growth condition.

In this review, we focus on the effects of specific root metabolites on nematodes ranging from plant parasitic to soil borne free-living nematodes. In **Tables 1**–**3** we present root exudates and specific root compounds that interact with plant parasitic nematodes and describe their effects on nematode taxa.

**Table 1:** Root metabolites affecting nematode movement.

Plant species/ synthetic chemicals	Root metabolites	Assay concentrations	Test system	Nematodes affected	Effect	Reference
^1^Tomato *Solanum lycopersicum*	Methyl salicylate	40, 80, 160 ng/µl dissolved in hexane, hexane as negative control	Olfactory assay with sterilized sand	*Meloidogyne incognita*	Attractant	(Murungi et al., 2018)
^1^Spinach *Spinacia oleracea*	2-isopropyl-3-methoxypyrazine, tridecane	40, 80, 160 ng/µl dissolved in hexane, hexane as negative control	Olfactory assay with sterilized sand	*M. incognita*	Attractant	(Murungi et al., 2018)
^1^Pepper *Capsicum annuum*	Methyl salicylate, α-pinene, limonene and tridecane	20, 40, 80 ng/µl µl dissolved in hexane, hexane as negative control	Olfactory assay with sand	*M. incognita*	Attractant	(Kihika et al., 2017)
^3^Synthetic chemicals	Isoamyl alcohol, 1-butanol, and 2-butanone	Dissolved in sterile ethanol (0.05% v/v) final concentration NA, water as negative control	*In vitro* assay	*M. incognita*	Attractant	(Shivakumara et al., 2018)
^3^Synthetic chemicals	Salicylic acid	20, 50, 100, 200 µg/ml; controls consisted of the compound solvent (0.5% DMSO, 2.5 mM NaOH or distilled water), 1% acetic acid as a repellent and 0.5 M CaCl_2_ as an attractant control.	*In vitro* assay	*M. incognita*	Attractant	(Wuyts et al., 2006)
^1^Tomato *S. lycopersicum*	Zeatin	4, 15.6, 62.3, 250, 1000 ng/μl dissolved in 2% DMSO; 2% DMSO as negative control and 100 ng/μlmethyl salicylate as positive control	Sand assay	*M. incognita*	Attractant	(Kirwa et al., 2018)
^3^Synthetic chemicals	Dopamine	20, 50, 100, 200 µg/ml; controls consisted of the compound solvent (0.5% DMSO, 2.5 mM NaOH or distilled water), 1% acetic acid as a repellent and 0.5 M CaCl_2_ as an attractant control.	*In vitro* assay	*Radophulus similis*	Attractant	(Wuyts et al., 2006)
^1^Tomato *S. lycopersicum*	L-ascorbyl 2, 6-dipalmitate; 2, 6-Di-tert-butyl-p-cresol; dibutyl phthalate and dimethyl phthalate	0.5mM, 1.1mM, 2.2mM dissolved in 1% ethanol, 1% ethanol as control	*In vitro* assay	*M. incognita*	Dimethyl phthalate repellent, all four compounds nematicidal	(Yang et al., 2016)
^1^Castor *Ricinus communis*	Palmitic acid and linoleic acid	0.5,1, 2, 4 mM dissolved in methanol, methanol as control	*In vitro* assay	*M. incognita*	Repellent	(Dong et al., 2018)
^1^Tomato *S. lycopersicum*	Quercetin	4, 15.6, 62.3, 250, 1000 ng/μl dissolved in 2% DMSO; 2% DMSO as negative control, 100 ng/μl methyl salicylate as positive control	Sand assay	*M. incognita*	Low concentration act as attractant and higher concentration as repellent	(Kirwa et al., 2018)
^3^Synthetic chemicals	1-octanol	Dissolved in sterile ethanol (0.05% v/v), final concentration NA, water as negative control	*In vitro* assay	*M. incognita*	Repellent	(Shivakumara et al., 2018)
^2^Marigold *Tagetes patula*, ^2^Pepper *Capsicum annuum*, and ^2^Soybean *Glycine max*	Root exudate compounds	Extract doses in bioassays expressed as mg of tip section (mg eq) volume^−1^, 1 to10 mg equivalent dispenser^-1^; double distilled water as control	*In vitro* assay	*M. incognita* and *Heterodera glycine*	Repellent to root knot nematode and attractant to cyst nematode	(Wang et al., 2018)
^2^Pea *Pisum sativum*, ^2^Snap bean *Phaseolus vulgaris*, and ^2^Alfalfa *Medicago sativa* cv. Thor, cv. Moapa 69, cv. Lahonton	Root tip exudates	Border cells, root tip exudates, and border cells + root tip exudates with water as control	*In vitro* assay	*M. incognita*	Repellent to J2s; induced quiescence response, > 80% of the nematodes lost motility	(Zhao et al., 2000)
^1^Tomato *S. lycopersicum* and ^1^Rice *Oryza sativa*	Small lipophilic molecules	Dissolved in 0.01% ethanol, final concentration NA, 0.01% ethanol and distilled water as control	*In vitro* assay	*M. incognita and Meloidogyne graminicola*	Repellent	(Dutta et al., 2012)
^3^Synthetic chemicals	p-coumaric acid, caffeic acid, ferulic acid, kaempferol, quercetin, myricetin	20, 50, 100, 200 µg/ml; controls consisted of the compound solvent (0.5% DMSO, 2.5 mM NaOH or distilled water), 1% acetic acid as a repellent and 0.5 M CaCl_2_ as an attractant control		*M. incognita*	Repellent	(Wuyts et al., 2006)
^3^Synthetic chemicals	Protocatechuic acid, umbelliferone, caffeic acid, ferulic acid, luteolin, daidzein, genistein, kaempferol, quercetin, myricetin	20, 50, 100, 200 µg/ml; controls consisted of the compound solvent (0.5% DMSO, 2.5 mM NaOH or distilled water), 1% acetic acid as a repellent and 0.5 M CaCl_2_ as an attractant control		*R. similis*	Repellent and nematicidal	(Wuyts et al., 2006)
^2^Potato *Solanum tuberosum*	Unknown volatile metabolites in root exudates	NA	Bioassay in sand	*Globodera pallida*	Attractants	(Farnier et al., 2012)
^3^Synthetic chemicals	Trans-cinnamic acid; p-coumaric acid	Trans-cinnamic acid 270 µM; p-coumaric acid 240 µM, water as control	*In vitro* assay	*M. incognita*	Repellent	(Fleming et al., 2017)
^3^Synthetic chemicals	Salicylic acid, ethephon, vanillic acid, gibberellic acid, indole-3-acetic acid, 6-dimethylallylamino purine, mannitol, arginine and lysine	Salicylic acid 100 µM; ethephon 1, 10, 50 µM; vanillic acid 240 µM; gibberellic acid 115 µM; indole-3-acetic acid 230 µM; 6-dimethylallylamino purine 200 µM; mannitol 5 mM; arginine 5 mM; and lysine, 5 mM; water as control	*In vitro* assay	*M. incognita*	Attractants	(Fleming et al., 2017)
^3^Synthetic chemicals	Salicylic acid, methyl jasmonate, ethephon, indole-3-acetic acid, mannitol	Salicylic acid 100 µM; methyl jasmonate 100 µM; ethephon 50 µM; indole-3-acetic acid 230 µM; mannitol 5 mM, water as control	*In vitro* assay	*G. pallida*	Attractants	(Fleming et al., 2017)
^1^Crown daisy*Glebionis coronaria*	Lauric acid	0.5, 1.0, 2.0, and 4.0mM dissolved in methanol, methanol as control	*In vitro* assay	*M. incognita*	Lauric acid (0.5, 1.0, 2.0 mM) acts as attractant and lethal trap and also act as repellent (4mM)	(Dong et al., 2014)
^2^Soybean *Glycine max* and ^2^Arabidopsis *Arabidopsis thaliana*	Metabolites of ethylene pathway	NA	*In vitro* assay	*Heterodera glycines*	Ethylene (ET)-synthesis inhibitor and ET-insensitive mutations attractant to cyst nematode	(Hu et al., 2017)
^2^Thale cress *Arabidopsis thaliana*	Metabolites of ethylene pathway	NA	*In vitro* assay	*Heterodera schachtii*	Ethylene treated Arabidopsis more attractive to nematodes	(Kammerhofer et al., 2015)
^2^Thale cress* Arabidopsis thaliana* and ^2^Tomato *S. lycopersicum*	Metabolites of ethylene pathway	NA	*In vitro* assay	*Meloidogyne hapla*	Ethylene (ET)-synthesis inhibitor and ET-insensitive mutations attractant while ET-overproducing mutants less attractive	(Fudali et al., 2012)
^2^Tomato *S. lycopersicum* and ^2^barrel clover *Medicago truncatula*	Metabolites of ethylene pathway	NA	*In vitro* assay	*M. hapla, Meloidogyne javanica*, and *M. incognita*	Mutants defective in ethylene signaling of both hosts were found to be more attractive compared to wild type	(Čepulyte et al., 2018)

^1^study on effect of both plant and specific metabolites detected in root exudates of the plant, ^2^study on effect of plant, ^3^study on effect of synthetic compound on nematodes, NA indicate not applicable.

**Table 2:** Nematicidal and nematode inhibitory root metabolites.

Plant species/ synthetic chemicals	Root metabolites	Assay concentrations	Test system	Nematodes affected	Effect	Reference
^3^Synthetic chemicals	Purified VOC dimethyl disulfide (DMDS) and 3-pentanol	DMDS (100, 200, 300, 400, 500, 600, 700 ppm), 3-pentanol (200, 400, 600, 800, 1000, 1200 ppm), water as control	*In vitro* assay	*M. incognita*	Toxicity to J2s and reduced egg masses and gall formation	(Silva et al., 2018)
^3^Synthetic chemicals	DMDS and 3-pentanol	500 ppm and 1000 ppm, water as control	*In vitro* assay	*M. incognita*	Toxicity to eggs and J2s and reduced the number of galls and egg masses	(da Silva et al., 2019)
^3^Synthetic chemicals	DMDS	Actual DMDS concentration N/A	Field trials and pot experiment	*M. incognita, M. hapla, Pratylenchus penetrans, Heterodera* sp.*, H. carotae, Globodera* sp.	Nematicidal	(Coosemans, 2005; López-Aranda et al., 2009; Curto et al., 2014; Leocata et al., 2014; Zanón et al., 2014; Myrta et al., 2018)
^1^Arugula *Eruca sativa*	Erucin	1 to 1000 ppm, water and mixture of methanol and aqueous Tween 20 at 0.3% (v/v) as control	*In vitro* assay	*M. incognita*	Nematicidal	(Aissani et al., 2015)
^1^Siam weed *Chromolaena odorata*	1,2-dehydropyrrolizidine alkaloids	7, 70, 350 ppm, water as control	*In vitro* assay	*M. incognita*	Nematicidal	(Thoden et al., 2007)
^1^Smooth Crotalaria *Crotalaria pallida*	Root exudates compounds - Proteinaceous papain inhibitor	Proteinaceous papain inhibitor 0.2, 0.5, 1 μg/μL, water and buffer as control	*In vitro* assay	*M. incognita*	Nematostatic and nematicidal	(Andrade et al., 2010)
^3^Synthetic chemicals	Butyric, caprylic, capric, lauric, myristic, palmitic, and oleic acids	100, 1000, 2000 μM dissolved in Tween-80/dimethyl sulfoxide/acetone/water, Tween-80/dimethyl sulfoxide/acetone/water as control	*In vitro* assay for mortality assessment, planted pot experiment for reproduction assessment	*M. incognita*	Mortality of J2s and reduced reproduction	(Zhang et al., 2012)
^3^Synthetic chemicals	Glucosinolates plus myrosinase, hydrolysis products gluconasturtiin, glucotropaeolin, glucoerucin, sinigrin, glucoraphasatin, gluconapin, glucoiberin, sinalbin, epi-progoitrin, glucoraphanin, glucoconringiin	0.0025 to 25 mM, phosphate buffer as control	*In vitro* assay	*M. incognita*	Nematicidal	(Lazzeri et al., 1993; Lazzeri et al., 2004)
^3^Synthetic chemicals	Glucosinolates plus myrosinase	Dehydroerucin, gloconapin, glucotropeolin, sinigrin; 0.005, 0.05, 0.5% (w/v), phosphate buffer as control	*In vitro* assay	*M. incognita*	Nematicidal	(Lazzeri et al., 1993; Lazzeri et al., 2004)
^1^Rattlepod *Crotalaria* sp. and ^1^Ragwort *Senecio jacobaea*	1,2-dehydropyrrolizidine alkaloids	1, 0.1 and 0.01 mg/ml, water as control	*In vitro* assay	*M. incognita, H. schachtii, P. penetrans, Phasmarhabditis hermaphrodita, Rhabditis* sp.	Nematicidal and ovicidal	(Thoden et al., 2009a)
^1^Alfalfa *Medicago sativa*	Medicagenic acid	125, 250, 500, 1,000 μg/ml	*In vitro* assay	*M. incognita* and *Xiphinema index*	Nematicidal	(D’Addabbo et al., 2011)
^3^Synthetic chemicals	Sinigrin, glucosinalbin, gluconapin, epi-progoitrin, erucin, glucoiberin	0.05, 0.30, 1.0 and 2.0 mg/ml, water as control	*In vitro* assay	*H. carotae* and *X. index*	Nematicidal	(Avato et al., 2013)
^1^Rye *Secale cereale*	Benzoxazinoids (DIMBOA, DIBOA, MBOA and BOA	0, 0.9, 4.5, 9, 22.5, 45, 67.5, and 90 mg/ml, deionized water as control	*In vitro* assay	*M. incognita* and *Xiphinema americanum*	Nematicidal	(Zasada et al., 2005)
^1^Plume poppy *Macleaya cordata*	Sanguinarine, chelerytherine and allocryptopine	1, 5, 10, 25, 50, 100, 200 µg/ml, dissolved in 5% of acetone, 5% of acetone as control	*In vitro* assay	*M. incognita, Caenorhabditis elegans* and *Bursaphelenchus xylophilus*	Nematicidal	(Kui et al., 2015)
^3^Synthetic chemicals	Thymol, benzaldehyde	Thymol (0, 50, 100, and 150 ppm) to soil in combination with benzaldehyde (0, 50, and 100 ppm), dissolved in ethanol, ethanol as control	Planted pot experiment	*Meloidogyne areneria, Heterodera minor, Paratrichodorus minor,* Dorylaimoid nematode	Reduced root galling, cyst formation, suppressed population growth	(Soler-Serratosa et al., 1996)
^3^Synthetic chemicals	Geraniol	62.5, 125, 250, 500 and 1000 ppm dissolved in water with ethanol and Tween-20, distilled water and water with ethanol and Tween-20 as control	*In vitro* assay	*M. javanica*	Nematicidal	(Nasiou and Giannakou, 2018)
^3^Synthetic chemicals	Me pelargonate and ethylene glycol pelargonate	0.2, 0.4, 0.8, 1.6, 3.2, 6.4, 12.5, 25, 50, 100 µl active ingredient l^-1^, deionized water as control	*In vitro* assay	*M. javanica* and *H. glycines*	Reduced number of galls and cysts	(Davis et al., 1997)
^3^Synthetic chemicals	α-terthienyl and gallic and linoleic acids	0.00125, 0.0025, 0.005, 0.01 mg/ml, dissolved in DMSO, DMSO and water as control, carbufuran as positive control	*In vitro* assay	*Heterodera zeae*	Nematicidal	(Faizi et al., 2011)
^3^Synthetic chemicals	*Mixture of* glucosinolates and active myrosinase	0.00125, 0.0025, 0.005, 0.01 mg/ml glucosinolate stock, myrosinase 0.05, 0.3, 1.0 mg/ml, dissolved in DMSO, water and DMSO as controls	*In vitro* assay	*Globodera rostochiensis*	Nematicidal	(Buskov et al., 2002; Aires et al., 2009)
^2^Sticky nightshade *Solanum sisymbriifolium* and ^2^Nightshade *Solanum nigrum*	Root exudates compounds	NA	*In vitro* assay	*Pratylenchus goodeyi*	Nematicidal	(Gouveia et al., 2014)

^1^study on effect of both plant and specific metabolites in root exudates of the plant, ^2^study on effect of plant, ^3^study on effect of synthetic compound on nematodes, NA indicate not applicable.

**Table 3:** Root metabolites affecting nematode hatching.

Plant species/ synthetic chemicals	Root metabolites	Assay conditions and concentrations	Test system	Nematodes affected	Effect	Reference
^1^Welsh onion *Allium fistulosum*	4-hydroxybenzeneethanol	0.3, 0.6, 1.2, 2.4, 4.8, 9.6, and 19.2 mM dissolved in ethanol, sterile distilled water as control	*In vitro* assay	*M. incognita*	Hatching inhibitor	(Li et al., 2018)
^2^Tall fescue *Festuca arundinacea*	Root exudates compounds	Exudate concentrations 0.35, 0.70, 1.05, and 1.40% (w/v) diluted in sterile distilled water, sterile distilled water as control	*In vitro* assay	*M. incognita*	Hatching inhibitor	(Meyer et al., 2013)
^2^Sticky nightshade *Solanum sisymbriifolium* (cv. Sharp)	Root exudate compounds	Exudates of tested plants; root exudates of tomato and distilled water were used as controls, final concentrations NA	*In vitro* assay	Root knot nematodes	Hatching inhibitor of *M. arenaria, Meloidogyne chitwoodi, M. hapla* and *M. hispanica; J2s* not able to penetrate the plant and highly resistant	(Dias et al., 2012)
^2^Sticky nightshade *Solanum sisymbriifolium* (cv. Sis 4004)	Root exudate compounds	Exudates of tested plants; root exudates of tomato and distilled water were used as controls, final concentrations NA	*In vitro* assay	Root knot nematodes	Hatching inhibitor of *M. arenaria, M. hapla* and *M. hispanica; J2s* not able to penetrate the plant and highly resistant	(Dias et al., 2012)
*^2^Sticky nightshade Solanum sisymbriifolium* (cv. Pion)	Root exudate compounds	Exudates of tested plants; root exudates of tomato and distilled water were used as controls, final concentrations NA	*In vitro* assay	Root knot nematodes	Hatching inhibitor of *M. arenaria, M. hapla* and *M. javanica; J2s* not able to penetrate the plant and highly resistant	(Dias et al., 2012)
^1^Brown mustard *Brassica juncea*	2-propenyl isothiocyanate	0.002%	*In vitro* assay	*G. pallida*	Hatching inhibitor	(Brolsma et al., 2014)
^2^Potato *Solanum tuberosum*	Root exudate compounds	0.0001, 0.001, 0.01, 0.1, 1 mg/ml root leachate and water and buffer as control	*In vitro* assay	*G. rostochiensis* and *G. pallida*	Hatching stimulants and attractants	(Devine and Jones, 2003)
^3^Synthetic chemical	Picrolonic acid	0.4 to 4mM	*In vitro* assay	*Heterodera rostochiensis* (Syn. *Globodera rostochiensis*)	Hatching stimulant	(Clarke and Shepherd, 1966)
^1^Potato *Solanum tuberosum*	Alpha-solanine and alpha-chaconine, picrolonic acid, sodium metavanadate, sodium orthovanadate, and sodium thiocyanate	Dilution over a 10-concentration logarithmic series of 10 mg/ml stock solutions, water as control	*In vitro* assay	*G. rostochiensis* and *G. pallida*	Hatching stimulants	(Byrne et al., 2001)
^1^Tomato *S. lycopersicum* and ^1^Potato *Solanum tuberosum*	Solanoeclepin A	NA	NA	*G. rostochiensis* and *G. pallida*	Hatching stimulant	(Schenk et al., 1999)
^2^Sticky nightshade *Solanum sisymbriifolium* and ^2^Nightshade *Solanum nigrum*	Root exudate compounds	Cysts were soaked in tap water for 1 week and then exposed to 25 ml root diffusate	*In vitro* assay	*G. rostochiensis* and *G. pallida*	Hatching stimulants, reduced number of J2s per cyst and decreased population	(Scholte, 2000)
^2^Sticky nightshade *Solanum sisymbriifolium*	Root exudate compounds	Treatments included control containers with soil without plants, containers with soil with susceptible potato cv. Bintje and containers with soil with *S. sisymbriifolium*	Plant-soil greenhouse experiment	*G. pallida*	Hatching stimulants, reduced number of J2s per cyst and decreased population	(Timmermans et al., 2006)
^2^Sticky nightshade *Solanum sisymbriifolium* cvs Melody, Pion, Sis 4004 and Sis 6001	Root exudate compounds	Cysts were exposed to 5 mL root exudate of each plant cultivar, Potato (*S. tuberosum* cv. Désirée) root exudate as positive control, tap water as negative control.	*In vitro* assay	*G. rostochiensis* and *G. pallida*	Hatching stimulants; inhibit reproduction	(Dias et al., 2017)
^2^Nightshade *Solanum nigrum*	Root exudate compounds	Cysts exposed to root exudate solutions collected 3 weeks after emergence (1:1, 1:10, 1:100, or 1:1000 exudate to distilled water).	*In vitro* assay	*Globodera tabacum*	Hatching stimulants	(LaMondia, 1995)
^1^Soybean *Glycine max* and ^1^Kidney bean *Phaseolus vulgaris*	Glycinoeclepin A,	10^-11^-10^-12^ g glycinoeclepin A mL^-1^	*In vitro* assays	*H. glycines*	Hatching stimulants	(Fukuzawa et al., 1985; Masamune et al., 1987)
^2^Sunn hemp *Crotalaria juncea* and ^2^Showy rattlepod *Crotalaria spectabilis*	Root exudate metabolites	One milliliter of root leachate added to 1 ml of egg suspension (600 eggs/ml), water as control.	Hatching assessed *in vitro*; penetration and development assessed in pot experiment, population response assessed in field trial	*H. glycines*	Hatching stimulants; stop nematode development in roots	(Kushida et al., 2003; Warnke et al., 2008)
^3^Synthetic chemical	Picrolonic acid	0.1 to 4mM	*In vitro* assay	*H. glycines, H. tabacum, H. cruciferae*	Stimulated hatching except cabbage cyst nematode	(Clarke and Shepherd, 1966)

^1^study on effect of both plant and specific metabolites in root exudates of the plant, ^2^study on effect of plant, ^3^study on effect of synthetic compound on nematodes, NA indicate not applicable.

## Plant Parasitic Nematodes Navigate *via* Root Chemical Cues

Nematodes perceive their surrounding environment through chemosensory perception. Typically, plant parasitic nematodes locate their preferred host through root exudate signals (Bird, 2004). Several chemical gradients exist around physiologically active roots and it is likely that some chemicals constitute “long distance attractants”, which help nematodes migrate towards root occupied soil volumes, whereas “short distance attractants” may aid nematode navigation to individual roots of a host (Perry, 2005). Infective J2 larvae of root knot nematodes *Meloidogyne incognita* and *M. graminicola* take the most direct route to their preferred host; however, they take the longest route towards poor hosts, which indicates that specific root metabolites act as attractants and repellants, respectively, and influence the movement patterns of the nematodes to find their suitable host (Reynolds et al., 2011).

### Attractants

Under natural conditions, volatile compounds are long distance cues for infective root knot nematode J2 larvae location of suitable hosts. More locally in the root region, water soluble chemicals act as signaling cues (Curtis et al., 2009). For instance, *M. incognita* is able to perceive and utilize plant volatile organic compounds for host location (Kihika et al., 2017). Still, we know very little about the identity of compounds involved in nematode attraction to hosts, but recent studies have identified some host-elicited attractants (**Table 1**).

Five components [2-isopropyl-3-methoxypyrazine, 2-(methoxy)-3-(1-methylpropyl) pyrazine, tridecane, and α- and β-cedrene] were identified in the root-emitted volatiles of both tomato and spinach, while three others (δ-3-carene, sabinene, and methyl salicylate) were specific to tomato roots volatiles. In bioassays, 2-isopropyl-3-methoxypyrazine and tridecane attracted *M. incognita* J2 larvae to spinach roots, but methyl salicylate was more attractive to the J2s than these two compounds, and repeated experiments confirmed that methyl salicylate renders tomato roots more attractive to *M. incognita* than spinach roots (Murungi et al., 2018). Similarly, among *Capsicum annum*-emitted root volatiles methyl salicylate exerted the strongest positive chemotaxis of infective *M. incognita* J2 larvae, followed by pinene, limonene, tridecane, and 2-methoxy-3-(1-methylpropyl)-pyrazine (Kihika et al., 2017). Hence, two studies (Kihika et al., 2017; Murungi et al., 2018) identify methyl salicylate as the most significant volatile attractant of *M. incognita* in the investigated Solanaceous plants. In a bioassay, salicylic acid attracted *M. incognita*, and dopamine attracted *Radopholus similis* (Wuyts et al., 2006).

We have limited information about compounds that attract cyst nematodes. Unknown volatile metabolites in potato root exudates attracted J2 larvae of the potato cyst nematode *Globodera pallida* (Farnier et al., 2012). In a bioassay, ethephon, methyl jasmonate, salicylic acid, indole acetic acid, and mannitol showed positive chemotaxis of *G. pallida* J2s (Fleming et al., 2017). In *in vitro* nematode infection assays, *Arabidopsis* mutants with a strigolactone signaling pathway deficiency reduced attraction and invasion by the cyst nematode *Heterodera schachtii* compared to the wildtype plant (Escudero Martinez et al., 2019).

### Repellants

The identification of compounds that repel plant parasitic nematodes (**Table 1**) may be an important step towards better control measures. Second stage juveniles of three root knot nematodes (*Meloidogyne hapla, M. javanica*, and *M. incognita*), were highly attracted to root tips of both tomato plants and barrel clover (*Medicago truncatula*). However, ethylene signaling deficient mutants roots attracted more nematodes than the wild type (Čepulyte et al., 2018). Similarly, *Arabidopsis* in which ethylene synthesis was inhibited were more attractive to *M. hapla*, but ethylene-overproducing mutants roots were less attractive. Roots of an ethylene insensitive tomato mutant were also more attractive (Fudali et al., 2012). These examples suggest that either ethylene or products of ethylene-responsive pathways generally repel root-knot nematodes.

For cyst nematodes, the influence of ethylene is less clear-cut. Roots of soybean and *Arabidopsis* treated with ethylene synthesis inhibitor attracted more Soybean cyst nematodes (*Heterodera glycines*), and significantly more nematodes penetrated the roots of ethylene synthesis inhibited plants. On the other hand, ethylene insensitive mutants roots of *Arabidopsis* accessions were more attractive to *H. glycines* than the wild type (Hu et al., 2017). Ethylene-overproducing *A. thaliana* mutants roots were hypersusceptible to beet cyst nematode (*Heterodera schachtii*), and ethylene-insensitive mutants were less susceptible to *H. schachtii* (Wubben et al., 2001). Similarly, ethylene treated plant roots were more attractive to soybean cyst nematode and were infected much faster, resulting in a higher infection rate (Kammerhofer et al., 2015). Future studies should therefore aim to reveal, whether root-knot nematode repellence is governed directly by ethylene or by other compounds in ethylene responsive pathways. Further, more information on the impact of ethylene or ethylene pathways on cyst nematodes and other plant parasitic nematode taxa will disclose, if ethylene is a broad-spectrum repellant.

Still, most specific compounds have only been demonstrated to repel a single nematode taxon in a single plant species (**Table 1**). It is therefore premature to draw general conclusions on which repellents most efficiently repel different species of plant parasitic nematodes.

Some plant metabolites that efficiently repelled plant parasitic nematodes in assays without plants could be objects of further investigation. For instance, *Capsicum annum* (pepper) root derived thymol, either alone or combined with other root volatiles of *C. annum* induced negative chemotaxis of both root-knot, cyst and stubby root nematodes (Kihika et al., 2017). Some flavonoids also repel plant parasitic nematodes, but for these compounds, the effect appears to be more species dependent. For instance, the flavonoids kaempferol, quercetin, and myricetin repelled *Radopholus similis* and *Meloidogyne incognita*, but not *Pratylenchus penetrans.* Other flavonoids, e.g. luteolin, daidzein, and genistein repelled *R. similis*, but had no effects on and *M. incognita* and *P. penetrans* (Wuyts et al., 2006).

## Nematicidal/Inhibitory Root Compounds

Some plant taxa, e.g. *Tagetes* and Brassicaceae, are well-known for their production and release of nematode-defensive compounds (**Table 2**). Inclusion of plants with high contents of nematicidal or nematode inhibitory compounds in cropping systems as a sanitation strategy has thus received considerable research attention and is also applied in practice. Further, the application of purified nematicidal plant-derived compounds may be an efficient nematode management strategy (Zanón et al., 2014).

*Allium* species (e.g. leek, onion, and garlic) contain sulfur amino-acid precursors in their cytoplasm, which upon cellular degradation are broken down by the enzyme allinase to a new volatile organic compound, dimethyl disulfide (DMDS) (Haroutunian, 2015). Purified DMDS killed J2 juveniles and reduced egg masses and gall formation of *M. incognita* on tomato roots (Silva et al., 2018). DMDS is now available as a commercial biofumigant, which applied in tobacco field trials significantly reduced both *M. incognita* and *Heterodera* spp. infestation (Zanón et al., 2014). Similarly, DMDS was also effective against potato cyst nematodes and root knot nematodes on potato and tomato plants (Coosemans, 2005), *M. hapla* and *P. penetrans* on strawberry (López-Aranda et al., 2009), cyst nematode (*H. carotae*), and *M. incognita* on carrot (Curto et al., 2014), on tomato plants (Myrta et al., 2018), and on watermelon (Leocata et al., 2014).

Glucosinolates are one of the most frequently studied groups of defensive secondary metabolites in plants. Upon cellular disruption, e.g. wounding by nematodes, the thioglucoside linkage is hydrolyzed by endogenous enzymes (myrosinases), resulting in the formation of products (e.g. isothiocyanate, thiocyanate, nitrile, epithionitrile, oxazolidine-2-thione) that are active against herbivores and pathogens (Fahey et al., 2001; Lambrix et al., 2007; Santolamazza-Carbone et al., 2014). For instance, glucosinates purified from Brassicaceae (*Brassica napus, B. rapa, B. carinata, Lepidium sativum, Raphanus sativus,* and *Sinapis alba*) were not toxic to J2s of the cyst nematode *Heterodera schachtii* in their original form, but enzymatic hydrolysis products of glucosinolates (isothiocyanate, sinigrin, gluconapin, glucotropeolin, dehydroerucin) were lethal to the nematode (Lazzeri et al., 1993). Similarly, 11 glucosinolates and their degradation products did not affect J2s of the root knot nematode *M. incognita*, but myrosinase hydrolysis products (gluconasturtiin, glucotropaeolin, glucoerucin, and sinigrin) were highly toxic (Lazzeri et al., 2004). Other studies also report *that* glucosinolates are only lethal to the cyst nematode *Globodera rostochiensis* in the presence of myrosinase (Buskov et al., 2002; Aires et al., 2009).

Pyrrolizidine alkaloids (PAs) are secondary metabolites in different species within the Asteraceae, Boraginaceae, Fabaceae, Convolvulaceae, Orchidaceae, and Apocynaceae (Rizk, 1991; Trigo, 2011), notably *Crotalaria* spp., *Ageratum* spp. and *Senecio* spp. (Asres et al., 2004; Flores et al., 2009; Thoden et al., 2009b; Stegelmeier, 2011). PA was found toxic to the plant parasitic nematodes *Meloidogyne incognita*, *Heterodera schachtii* and *Pratylenchus penetrans* (Thoden et al., 2009a). In a bioassay, a PA [Loline (N-formylloline)] in root exudates of Tall fescue, was reported nematicidal to J2s of *Pratylenchus scribneri* (Bacetty et al., 2009). Likewise, PA-containing *Ageratum houstonianum* and *Senecio bicolor* inhibited *M. hapla* reproduction totally, but *M. hapla* reproduced on other PA-containing species (Thoden et al., 2009b; Vestergård, 2019). Hence, adoption of PA-containing plants for the management of plant parasitic nematodes must rely on careful selection of suitable species.

α-terthienyl, usually abundant in marigold (*Tagetes* spp.) tissue, is one of the most extensively studied nematicidal compounds (Morallo-Rejesus and Decena, 1982; Nivsarkar et al., 2001; Hamaguchi et al., 2019). Although the negative effect of *Tagetes* on plant parasitic nematodes is not always achieved in the field (Hooks et al., 2010), many trials demonstrated the mitigating potential of *Tagetes*. For instance, *T. patula* reduced *P. penetrans* densities for three consecutive years and alleviated damage to strawberries from *P. penetrans*, and these effects lasted longer than the effect of chemical soil fumigation (Evenhuis et al., 2004). Likewise, intercropping tomato plants with *T. patula* reduced *Meloidogyne* reproduction and root galling (Tringovska et al., 2015). Under *in vivo* conditions, root diffusate of marigold (*Tagetes patula* cv. Single gold) did not affect hatching pattern, migration and penetration of *M. chitwoodi* and *P. penetrans* compared to tomato roots, but reduced *M. chitwoodi* and totally prevented *P. penetrans* reproduction (Nježić et al., 2014). Other bioactive *Tagetes* compounds may be involved in nematode suppression (Hooks et al., 2010), but there is no doubt that α-terthienyl is a potent nematicide. For instance, α‐terthienyl exhibited 100% mortality of *Heterodera zeae* at concentrations of 0.125% after 24h (Faizi et al., 2011). The biological activity of α‐terthienyl increases greatly upon near-UV exposure, resulting in the production of biocidal singlet oxygen (Marles et al., 1992). When the nematode penetrates the root, α‐terthienyl is activated by root peroxidases in the absence of light (Gommers and Bakker, 1988), and it has therefore been assumed that α‐terthienyl is only effective *in planta* and not active in the soil (Hooks et al., 2010). However, recently it has been reported that α‐terthienyl actually was nematotoxic *ex planta* without photoactivation (Hamaguchi et al., 2019).

Root exudate benzoxazinoids, such as 2, 4-dihydroxy-7-methoxy-2*H*-1,4-benzoxazin-3(4*H*)-one (DIMBOA), mainly produced in rye and other cereals, have been found toxic against mixed stages of American dagger nematode *Xiphinema americanum* (Zasada et al., 2005). Rye cultivars with higher root concentrations of methoxy-substituted benzoxazinoids had the lowest numbers of *M. incognita* eggs. These cultivars were therefore suggested for soil incorporation as green manure to protect against root-knot nematodes (Zasada et al., 2007). In a greenhouse trial, soil infested with the root-knot nematode *Meloidogyne incognita* was treated with DIBOA (2,4-Dihydroxy-2H-1,4-benzoxazin-3(4H)-one) at concentrations ranging from 1.1 to 18 µg/g dry soil, and *M. incognita* egg production in cucumber roots decreased significantly at the highest concentration (Meyer et al., 2009).

## Plant Defense Elicited by Plant Parasitic Nematodes

Plant hormones are widely studied as defensive strategies against plant parasitic nematodes. The jasmonate (JA) plant hormones play a key role during early plant defense against the soybean cyst nematode *Heterodera schachtii* (Kammerhofer et al., 2015), the columbia root-knot nematode *Meloidogyne chitwoodi* (Vieira dos Santos et al., 2013), *M. incognita* (Fujimoto et al., 2011) and *M. hapla* (Gleason et al., 2016), the root lesion nematode *Pratylenchus neglectus* and the oat cyst nematode *Heterodera avenae* (Soriano et al., 2004). Jasmonate also cross talks with other plant hormones and defend the plants from nematode attacks. For instance, plants treated with Me-jasmonate and ethephon (an ethylene analogue) made plants more defensive against the rice root knot nematodes *Meloidogyne graminicola* compared to untreated plants (Nahar et al., 2011).

Low levels of salicylic acid (SA) may be sufficient for basal and Mi-1 resistance to root knot nematodes (Bhattarai et al., 2008). SA application induced resistance to the clover cyst nematode *Heterodera trifolii* in white clover (Kempster et al., 2001), and to *Meloidgyne incognita* (Molinari, 2016), *M. javanica* (Moslemi et al., 2016) and *M. chitwoodi* in tomato plants (Vieira dos Santos et al., 2013). Abscisic acid (ABA) plays a complex role in plant defense responses. While it promotes resistance in some plant–pathogen interactions, it enhances susceptibility in others (Lim and Lee, 2015). For instance, in one study, the reproduction of root knot nematode *M. incognita* on potato roots was much lower in ABA-sprayed plants compared to control plants (Karimi, 1995), whereas exogenous application of ABA on rice plants enhanced gall formation by *Meloidogyne graminicola* and did not impair nematode development (Kyndt et al., 2017). Similarly, exogenous ABA application reduced tomato plant resistance against *Meloidogyne javanica* (Moosavi, 2017). These varied responses to ABA application shows the complex role of ABA in plant defense against nematodes.

## Hatching Stimulation/Inhibition

Cyst nematodes (G*lobodera* spp. and *Heterodera* spp.) generally have a very narrow host spectrum, and as active infective juveniles only have limited storage energy they will starve and die within a relatively short period without access to a suitable host. However, the encysted, dormant eggs stay viable for years to decades, and are triggered to hatch and re-activate by host-specific hatching stimulants. Application of hatching stimulants in the absence of host plants is therefore a promising strategy for efficient reduction of cyst nematode populations. A number of hatching stimulants have been identified (**Table 3**), e.g. picrolonic acid, which induce hatching of *Heterodera rostochiensis* (Syn. *Globodera rostochiensis*), *H. glycines* and *H. tabacum* (Clarke and Shepherd, 1966), glycinoeclepin, which induces *H. glycines* hatching (Masamune et al., 1987), and solanoeclepin, sodium thiocyanate, alpha-solanine, and alpha-chaconine, which induce hatching of *G. pallida* and *G. rostochiensis* (Schenk et al., 1999; Byrne et al., 2001).

## Modes of Action of Root Metabolites in Nematodes

For most plant-derived metabolites we know very little about their molecular mode of action in nematodes. Here we present examples of the few available studies on the effect of root metabolites on plant parasitic nematode gene expression.

*Arabidopsis thaliana* root exudates were found to affect gene expression in *M. incognita* J2 larvae, prior to physical contact and penetration of the root. Sixty three candidate genes were identified, which were differentially expressed within one hour of exudate exposure, providing the evidence that root exudates induce changes in *M. incognita* gene expression (Teillet et al., 2013). Later it was demonstrated that tomato root exudates differentially upregulated four candidate parasitism genes, namely calreticulin (*crt-1*), *β*-1,4 endoglucanase-1 (*eng-1*), cathepsin L cysteine protease (*cpl-1*), fatty acid retinol binding protein (*far-1*), and venom allergen-like protein (*vap-1*) in preparasitic *Meloidogyne hispanica* J2s (Duarte et al., 2015). However, the identity of the exudate compounds that elicit enhanced expression of these genes is still unknown. Their disclosure could potentially lead to the breeding of varieties with low levels of parasitism gene activators and thus new and improved strategies against root knot nematode infection.

Plant parasitic nematodes secrete plant cell wall degrading enzymes in order to penetrate the host (Mitsumasu et al., 2015). The first evidence that plant cell wall components and host root exudates regulate the expression of genes encoding such enzymes was published recently (Bell et al., 2019). Hence, *Pratylenchus coffeae* treated with cellulose or xylan or with root exudates of host plants up-regulated the gene expression of β-1,4-endoglucanase (*Pc-eng-1*) or β-1,4-endoxylanase (*Pc-xyl*) respectively. The study also confirmed that the expression of these two genes is important for root penetration (Bell et al., 2019).

Host exudate induction of cyst nematode egg hatching obviously involves exudate activation of genes in the dormant juvenile cyst nematode. For instance, in hydrated *G. rostochiensis* cysts, 8 h of potato root exudate exposure, resulted in up-regulation of a gene encoding for a transmembrane metalloprotease. This enzyme is known to activate/inactivate peptide hormones and may be involved in a cascade of events leading to hatching. After 48 h of exudate exposure, *G*. *rostochiensis* had 278 differentially expressed genes, several of which are known effector genes (Duceppe et al., 2017).

These studies on root knot and root lesion cyst nematodes demonstrate that root exudates influence gene expression of pre-parasitic phase/early phase of nematodes, but which exudate components are involved in gene regulation remains to be disclosed.

We know very little about the molecular responses elicited by nematode attractants, repellents, and toxins within the nematode body, but several studies demonstrate that root exudates regulate the expression of *flp* genes. *flp* genes encode FMRFamide-like peptides, a diverse group of neuropeptides, involved in nematode feeding, reproductive and locomotive behavior, and thus play a pivotal role in nematode chemotaxis. For instance, low concentrations (0.5–2.0 mM) of lauric acid from crown daisy (*Chrysanthemum coronarium*) root exudates attract *Meloidogyne incognita*, while higher lauric acid concentration (4.0mM) repels the nematode. This response is probably elicited by lauric acid’s concentration-dependent regulation of *Mi-flp-18* gene expression (Dong et al., 2014). Moreover, two other active compounds, namely palmitic acid and linoleic acid derived from castor root exudates, was found to repel *M. incognita* and inhibited the expression of *Mi-flp-18* and *Mi-mpk-1* (mitogen-activated protein kinase) genes in a concentration-dependent manner (Dong et al., 2018). Silencing *G. pallida flp* genes (*Gp-flp-1, -6, -12, -14,* or *-18*) resulted in aberrant behavioral phenotypes, which further confirms that *flp* genes play key roles in motor function and suggests that *flp* gene silencing can be a novel plant parasite control strategy (Kimber et al., 2007). Furthermore, for *C. elegans* loss of *flp-1* and *daf-10* also disrupted different neurons in the neural circuits (Buntschuh et al., 2018).

Since marigold derived chemical α-terthienyl is expected to exert nematicidal action in the soil, a recent study investigated the molecular action of this chemical without photoactivation. This study revealed that α-terthienyl is nematicidal also in the dark, albeit the effect is higher when the compound is photoactivated. Further, it was established that α-terthienyl is an oxidative stress-inducing chemical that effectively penetrates the nematode hypodermis and suppresses *gst-4* (glutathione S-transferase) and *sod-1* (superoxide dismutase) gene expression. This results in restricted production of glutathione S-transferase and superoxide dismutase, which are necessary for nematode defense responses (Hamaguchi et al., 2019).

## Effects of Root Metabolites on Non-Target Nematodes

Due to their direct impact on crop yield and quality, agricultural researchers and practitioners pay more attention to and are more aware of plant parasitic nematodes than the many species of soil dwelling non-herbivorous nematodes. Nevertheless, non-herbivorous nematodes perform functions that are essential to natural as well as agro-ecosystems.

With protozoa, microbial feeding nematodes are the principal microbial grazers in terrestrial ecosystems. They regulate the size, activity, and functioning of bacterial and fungal populations (Ingham et al., 1985; Rønn et al., 2012; Thakur and Geisen, 2019). The most important impact of nematode microbial grazing is the enhanced turnover and mineralization of plant nutrients, notably nitrogen, and thus stimulation of plant growth. Further, because bacterial taxa vary in terms of food quality and ingestibility for nematodes (Bjørnlund et al., 2012) nematode grazing changes the composition of bacterial communities (Xiao et al., 2014). Likewise, the quality as food for fungal feeding nematodes varies between fungal species (Chen and Ferris, 1999), and fungal feeding nematodes preferentially select certain fungal species (Ruess et al., 2000). Thus, nematode grazing on root-associated microorganisms probably modulates the plant-microbiome functional interactions.

Entomopathogenic nematodes, i.e. nematodes of the two genera *Heterorhabditis* and *Steinernema* are harmless to plants and under some circumstances even plant beneficial. *Heterorhabditis* spp. and *Steinernema* spp. are closely associated with species of insect lethal *Protorhabdus* and *Xenorhabdus* bacteria. The nematode enters the body cavity of the susceptible insect larvae, where the associated bacteria are released, multiply and eventually kill the insect. Bacteria growing on the insect cadaver then serve as food for the nematodes (Poinar and Grewal, 2012). Given the right conditions, entomopathogenic nematodes reduce the abundance of root detrimental insect larvae (Toepfer et al., 2009). Because non-herbivorous nematodes execute a variety of central functions in terrestrial systems, it is relevant to consider, if plant metabolites with adverse effects on plant parasitic nematodes similarly reduce the survival or performance of non-target nematodes.

Marigold (*Tagetes patula* cv. Single Gold) root exudates did not influence the migration rate of dauer juveniles of the entomopathogenic nematode *Steinernema feltiae* towards *Galleria mellonela* larvae. Even exposing dauer juveniles of *S. feltiae* for 24 hours to marigold root diffusate resulted in higher penetration rate of EPN compared to soil leachate (Nježić et al., 2010). In a bioassay, germinated seeds of marigold attracted *Steinernema carpocapsae*. Neither did aqueous root extracts of marigold adversely affect EPN infectivity, but synthetic α-terthienyl at concentrations of 20 and 40ppm significantly reduced the numbers of nematodes that infected insect hosts. This indicates that higher doses of this chemical may affect entomopathogenic nematodes (Kaya and Kanagy, 2010). *Caenorhabditis elegans*, a bacterial feeding nematode, was as sensitive as *Meloidogyne incognita* and 10 times more sensitive than *Pratylenchus penetrans* to α-terthienyl *in vitro* (Kyo et al., 1990; Hamaguchi et al., 2019).

In field trials, McSorley et al. (2009) and Wang et al. (2011b) assessed soil abundances of bacterial, fungal- and omnivorous/predatory nematodes as well as oribatid mites, predatory mites, and collembola after sunn hemp (*Crotalaria juncea*) and marigold cover crops and a fallow period. The comparison to a fallow treatment is not ideal for the evaluation of potential negative effects on microbial feeding nematodes and decomposer microarthropods, as the input of organic substrate for saprotrophic organisms is of course considerably lower in fallow than planted systems. It is therefore not surprising that sunn hemp mulching temporarily increased densities of bacterial and fungal feeding nematodes and microarthropods compared to fallow soil. The densities in marigold planted plots were as low as in the fallow plots at all sampling times, and could, although not unequivocally suggest that marigold prevented growth of microbial feeding nematodes. However, in other field experiments, densities of non-herbivorous nematodes were higher in marigold-planted than fallow plots and comparable to compost treated soil (Wang et al., 2011a; Korthals et al., 2014). Hence, the results from the limited number of field and *in vitro* studies on non-target effects of marigold root on non-herbivorous nematodes vary from negative effects over no effects to positive effects. Rigorously controlled experiments including the assessment of realistic and super-realistic concentrations of marigold metabolites on single nematode species and mixed communities are needed to reach more firm conclusions on their significance for the compositon and functioning of non-target nematode communities in practice.

The root exudates of green pea (*Pisum sativum*) induced reversible quiescence in all EPN species (*Heterorhabditis bacteriophora*, *H. megidis*, *Steinernema feltiae* and *S. carpocapsae)* tested. However, this response was concentration dependent, and diluted root exudates did not induce quiescence, but enhanced EPN activity and insect infectivity. The diluted root exudates still reduced the activity of the soybean cyst nematode *Heterodera glycines* and the root-knot nematode *Meloidogyne incognita* (Hiltpold et al., 2015). It is extremely difficult to determine which root exudate concentrations the nematodes are exposed to *in vivo*, but the authors presume that rhizosphere concentrations are below the level for quiescence induction (Hiltpold et al., 2015).

Soil incorporation of *Brassica carinata* reduced root-knot nematode *M. chitwoodi*, but also disrupted the ability of entomopathogenic nematodes *S. feltiae* and *S. riobrave* to control Colorado potato beetles (*Leptinotarsa decemlineata*). This study exposes the challenges of integrating biofumigation and biocontrol approaches in managing plant parasitic nematodes and other pests (Henderson et al., 2009). In a long-term field trial, incorporation of biofumigant Brassicaceae neither reduced plant parasitic nor total nematode abundances, but, as could be expected from the increased input of dead plant material to the soil, total densities of nematodes increased moderately (Korthals et al., 2014). Similarly, the biofumigant yellow mustard had none to slightly negative effects on plant parasitic nematodes and none to slightly positive effects on microbial feeding, omnivorous and predatory nematodes (Valdes et al., 2012).

Testing the effects of four different PAs, Thoden et al. (2009a) found that the mobility of bacterial feeding *Rhabditis* sp. was unaffected after 20 h exposure, but after a week’s exposure, especially one PA (monocrotaline) reduced *Rhabditis* sp. mobility. Further, monocrotaline repelled *Rhabditis* sp. Another PA (heliotrine) reduced *Rhabditis* sp. development and reproduction. The slug- and snail infecting nematode *Phasmarhabditis hermaphrodita* was completely unaffected by PAs (Thoden et al., 2009a; Thoden et al., 2009b). Further, it has been shown that the use of PA‐producing *Crotalaria* species as soil amendment increases the abundance of free‐living nematodes (Wang et al., 2007). In banana orchard, sunn hemp (*Crotalaria juncea* L.) thus consistently suppressed the population of the plant parasitic nematode *Radopholus similis*, while supporting the highest numbers of beneficial nematodes (bacterivorous, fungivorous, omnivorous, and predaceous species) (Henmi and Marahatta, 2018).

## Application of Root Metabolites in Nematode Management—Challenges and Opportunities

A considerable number of bioactive root compounds with documented effect on the behavior, development or even survival (**Tables 1**–**3**) on plant parasitic nematodes have been identified. Potentially, this knowledge may be applied in practical control or management of plant parasitic nematodes. However, in most cases, the effects were only tested on a single nematode species, which is only natural given that the field is still at an early explorative stage. As compounds that affect a wider spectrum of plant parasitic nematode species and other pest organisms will of course be most interesting in practice, investigations of the effects on a broader spectrum of nematode taxa are highly pertinent. On the other hand, there is the risk that metabolites that are active against a broad spectrum of plant parasitic nematodes will also have unwanted negative effects on non-parasitic nematodes. Given the importance of microbivorous and entomopathogenic nematodes for nutrient turnover and the control of root herbivorous insects, respectively, priorities should be given to metabolites that are harmless to or even beneficial for non-target nematodes.

The ability to identify and find suitable hosts is essential for all plant parasitic nematodes. Thus, the nematodes navigate and differentiate between different plant species aided by attractant and repellent plant metabolites. A range of exudate metabolites have been identified as attractant of *Meloidogyne incognita* and a few as attractants of *Pratylenchus* and cyst nematode species. Potentially, intercropping susceptible crops with nematode-resistant or even nematode-suppressive highly attractant plants can alleviate damage of crop plants (Dong et al., 2014). We therefore foresee that continuous efforts aimed at identifying and screening for nematode chemical attractants exuded by plant species with additional desirable intercropping properties will contribute to improved management of plant parasitic nematodes.

*Vice versa*, the identification of quite a few compounds that repel several species of plant parasitic nematodes is of course interesting. Selection and breeding of plant cultivars that release high levels of repellents could prove a promising strategy for reduced nematode infection levels. With this perspective, it is interesting that repellent compounds have been identified in economically important host plants, where root knot nematodes are particularly problematic, e.g. tomato plants (Yang et al., 2016; Kirwa et al., 2018). In horticultural production, grafting is becoming a common alternative to lengthy breeding for pathogen resistance. Aboveground parts of cultivars with desirable traits, e.g. high yields and/or high fruit quality are grafted on rootstocks of close relatives that are resistant to specific soil-borne pests or pathogens, e.g. nematodes (Kawaide, 1985; Oka et al., 2004; Galatti et al., 2013; Thies et al., 2015). We propose that grafting on highly nematode repellent rootstocks could be a similar fast track to reduce yield losses caused by nematodes.

The list of nematicidal root compounds is long, and again, many were only tested in a single study. However, some compounds such as lauric acids, DMDS, pyrrolizidine alkaloids (PAs), α-terthienyl and products of myrosinase-catalyzed hydrolysis of glucosinolates have proved lethal against both root knot, cyst and lesion nematodes. Accordingly, plants with high levels of some of these compounds are integrated in strategies for nematode control. In practice, the efficacy of crops that produce nematicidal root metabolites can be quite unpredictive. For instance, the myrosinase-catalyzed hydrolysis of glucosinolates must be induced by cell wall disruption such as insect or nematode attack, or during the degradation of dead plant parts. Further, myrosinase activity is temperature-dependent (Ploeg and Stapleton, 2001; Lopez-Perez et al., 2005). Whether the elicited production and release to the rhizosphere soil is sufficient to significantly reduce populations of plant parasitic nematodes may thus be very context-dependent. The application of purified DMDS, the nematicide produced after enzymatic conversion of sulfur amino-acid precursors in *Allium* species (Zanón et al., 2014; Haroutunian, 2015) demonstrates that better and more reliable nematode control may be obtained by direct application of the bioactive phytochemical.

The long persistence time for encysted eggs poses a special challenge for the control of cyst nematodes. To our knowledge, the potential for applying hatching stimulants for cyst nematode control or eradication remains to be investigated. However, inclusion of hatching-stimulating non-host plants in crop rotation schemes or even termination of host crops before cyst nematode reproduction is a way to reduce the density of persistent cysts in infected soil (Scholte, 2000; Dandurand and Knudsen, 2016). Field trials, where *Solanum sisymbriifolium* (sticky nightshade) induce hatching of *Globodera*, which are unable to fulfill their life cycle on *S. sisymbriifolium*, demonstrate that hatching induction in the absence of susceptible hosts efficiently reduce the density of resting, viable cysts in the soil (Scholte and Vos, 2000).

For the majority of specific root compounds their effects on nematodes were demonstrated *in vitro* (**Tables 1**–**3**). *In vitro* experiments are obviously necessary to provide conclusive evidence for the effect of candidate compounds. Meanwhile, the extrapolation of results obtained *in vitro* to rhizosphere and *in planta* conditions is not straightforward. For many compounds, information about their concentrations in roots, and in particular in rhizosphere soil, is sparse, and the concentration in the rhizosphere may easily be 1000 times lower than in the roots (Kudjordjie et al., 2019). We thus emphasize that effects detected at low concentrations, i.e. µM, ppm or lower, in assays covering a concentration gradient are more likely to reflect mechanisms that are relevant under realistic conditions.

It is evident that the physicochemical complexity of soil systems, be it in the greenhouse or in the field, can alter or altogether eliminate the effects exposed *in vitro*. For instance, NPK fertilization reduces PA concentrations in *Senecio* spp. roots (Hol, 2011) suggesting that plants regulate the production of defense compounds according to nutrient availability. Other abiotic factors such as soil moisture and structure modulate the diffusion and thus distribution of plant-derived volatile compounds in the soil matrix (Hiltpold and Turlings, 2008), and nematode responses to volatile cues therefore probably also depend on these factors. Hence, it will be interesting to see the outcome of more *in vivo* studies, e.g. assessing the impact of plant mutant lines that are impaired in or over-expressing specific metabolic pathways in different soil types and variable abiotic conditions.

Further, the production of many compounds that interfere with nematode performance are elicited by the presence of other above- or belowground herbivores. For instance, root exudates of potato plants exposed to the aphid *Myzus persicae* reduced egg hatching and interfered with cyst gene expression in *Globodera pallida* (Hoysted et al., 2018). Clearly, when plant chemical defense belowground depends on plant interactions with aboveground organisms it becomes difficult to forecast and rely on the efficacy of the belowground defense in practice. Moreover, symbionts also appear to regulate production of defense compounds; e.g. arbuscular mycorrhizal fungi reduced benzoxazinoid concentrations in wheat roots, while root infection by *Pratylenchus neglectus* was 47%–117% higher on mycorrhizal than non-mycorrhizal plants (Frew et al., 2018).

The biological complexity of soil systems is immense, and as such, root metabolites interact with multiple different soil organisms. Hence, most of the signaling and nematicidal compounds mentioned in this review also affect other pathogens. For instance, methyl salicylate and salycilic acids play multiple roles in plant defense against a long range of pathogens (Hammerbacher et al., 2019; Maruri-Lopez et al., 2019), and DIMBOA and other benzoxazinoids are also antagonistic towards insect pests and fungal pathogens (Fomsgaard et al., 2004). Plants that produce compounds that are antagonistic towards multiple pests and pathogens may be particularly valuable components in sanitation strategies.

The inevitable microbial turnover of exuded metabolites challenges the extrapolation of results from *in vitro* studies to soil conditions. For instance, while DIBOA proved an efficient nematicide in aqueous assays, the fast microbial turnover and possible absorption to soil particles reduced the nematicidal effect in soil (Meyer et al., 2009). Hence, microbial degradation may inactivate or reduce the activity of biocidal compounds in soil.

To complicate matters even more, the effects of some plant-derived chemicals vary between nematode species. Ethylene generally repels root knot nematodes, whereas at least some cyst nematodes are attracted by ethylene. Hence, in fields infested with both types of nematodes ethylene may on one hand reduce root-knot nematode infection, but on the other hand release cyst nematodes from competition from root-knot nematodes. Clearly, most research on root metabolite effects on nematodes has focused on the nematodes that are most damaging to economically important crops; i.e. root knot nematodes, particularly *M. incognita*, cyst nematodes and root lesion nematodes (Jones et al., 2013), and we know very little about the effects of root metabolites on ectoparasitic nematodes. Endo- and ectoparasitic nematode species may compete and dominate under different environmental conditions (Brinkman et al., 2004; Vestergård et al., 2004), and it is therefore worth considering if strategies targeting endoparasites can enhance populations of and crop damages exerted by ectoparasitic nematodes.

## Concluding Remarks

With the current and past withdrawal of chemical nematicides, insights on root chemical impacts on plant parasitic nematodes are important contributions to alternative strategies for plant parasitic nematode management. This review exposes that a diverse array of root chemicals across a range of plant taxa are potentially involved in nematode host location, egg hatching, and survival. Some of this insight is already exploited in practice, e.g. the use of nematicidal plants or application of purified plant-derived nematicides, and the use of egg hatching stimulating plants for cyst nematode management. However, we believe that there are opportunities for improved exploitation of root metabolites for nematode management. Many plant metabolites that have clearly proved bioactive under highly controlled laboratory conditions are less reliable in the field. The discrepancy between *in vitro* and *in vivo* efficacy may reflect that compounds were tested at unrealistic concentrations in the lab, that soil physicochemical factors reduce their activity, that they are inactivated by fast microbial degradation etc. There is therefore a need for experimental investigations to bridge the gap between highly controlled laboratory experiments and realistic field conditions. Such experiments, e.g. plant-soil mesocosm experiments, should aim to clarify the fate and activity of key metabolites in the rhizosphere as a function of e.g. soil texture, structure, pH, temperature, and microbial activity to facilitate better prediction of the bioactive efficacy in different real-world contexts.

Within the last five years, the first studies to establish how root chemicals regulate genetic expression in plant parasitic nematodes have been published. We foresee that further disclosures of nematode molecular responses to specific root metabolites, and thus in-depth understanding of their modes of action will help us predict which compounds hold the largest potential for efficient control or management of plant parasitic nematodes.

In general, we propose to focus further developments on nematicidal plant species and compounds with biocidal activity against a broad spectrum of parasitic nematodes as well as other pathogenic and pest organisms to facilitate integrated management of diverse plant pests. Hence, selection of and further breeding for cultivars that produce high levels of nematicidal or repellent metabolites can result in more nematode-resistant cultivars. In high value horticultural crops, e.g. tomato cultures grafting on rootstocks with high production of repellent or nematicidal metabolites could be a fast alternative to the lengthy breeding process.

As non-herbivorous soil nematodes contribute to decomposition processes, inorganic nutrient availability and even control of herbivorous insect larvae, it is relevant to consider if metabolites that are toxic to plant parasitic nematodes exert negative effects on these related non-target organisms. Only a limited number of studies assessed how plant metabolites that repress plant parasitic nematodes affect non-target nematodes, and with varied outcome. Therefore, we cannot draw any general conclusions on the sensitivity of microbivorous and entomopathogenic nematode species or communities to root metabolites.

## Author Contributions

MS and MV contributed equally to the conception and preparation of the manuscript.

## Funding 

MS was funded by a grant from Aarhus University, and MV was supported by a starting grant from Aarhus University Research Foundation.

## Conflict of Interest

The authors declare the research was conducted in the absence of any commercial or financial relationships that could be construed as a potential conflict of interest.
